# Bioengineering Innovations in Global Dental Infection Control: Applications and Adaptations in Clinical Settings

**DOI:** 10.1016/j.identj.2024.12.011

**Published:** 2024-12-30

**Authors:** Abdullah Khalid Amer AlSaiari, Mohammad S. Alonazi, Nasser Muneer Alotaibi, Hamad AlQahtani, Waleed Masoud Alotaibi, Awatef Sliman Saleh Al Yhyha, Aqilah Aldarorah, Nawal Megad Alaklabi, Thuraya Ahmed Saleh Al-Obayli, Ohoud Abdulhadi Salha Alsaud, Latifa Abdulrahman Alosaimi, Manal Ghazi Al Beshi

**Affiliations:** King Salman Hospital, Ministry of Health, Riyadh, Saudi Arabia

**Keywords:** Bioengineering Innovations, Dental Infection Control, Antimicrobial Biomaterials, Sterilization Technologies, Personal Protective Equipment

## Abstract

**Introduction and Aims:**

Dental practices pose a high risk of microbial contamination due to frequent exposure to bodily fluids like saliva and blood. Bioengineering innovations have emerged as vital tools to enhance infection control in dental settings. This review aims to assess the global applications and effectiveness of these innovations, particularly focusing on antimicrobial biomaterials, sterilization techniques, and personal protective equipment (PPE).

**Methods:**

A systematic review was conducted across major databases to identify studies from 2000 to 2024 that examined bioengineering technologies used in dental infection control. Inclusion criteria included studies focusing on antimicrobial materials, PPE, or novel sterilization technologies. Data extraction followed PRISMA guidelines, focusing on study design, dental settings, and clinical outcomes related to infection control.

**Results:**

Nine studies met the inclusion criteria, covering diverse geographical regions, including Italy, Saudi Arabia, Brazil, and India. Antimicrobial agents like silver and zinc oxide nanoparticles, UV-C sterilization, and low-temperature plasma were found to significantly reduce microbial contamination. The pooled effect size across studies was 1.62 (95% CI: 1.41-1.82) with low heterogeneity (I² = 6.4%). However, barriers such as high costs and limited resource availability were highlighted, particularly in lower-income regions.

**Conclusion:**

Bioengineering innovations show significant potential for enhancing infection control in dental settings worldwide. While the technologies offer improved safety and hygiene, challenges related to cost and accessibility persist. Further research is needed to explore cost-effective and scalable solutions, particularly in resource-limited settings.

**Clinical Relevance:**

The integration of bioengineering technologies in dental practices can significantly improve infection control measures, reducing microbial contamination and enhancing safety for both patients and healthcare workers. These innovations hold promise for global adaptation, particularly in response to emerging public health challenges.

## Introduction

Infection control is a fundamental aspect of healthcare delivery, particularly in dental settings where procedures often involve exposure to blood, saliva, and other bodily fluids, which are potential vectors for infectious agents. Dental practices are considered high-risk environments for the transmission of infections due to the proximity between dental professionals and patients and the frequent use of aerosol-generating procedures. Consequently, ensuring the effective prevention of cross-contamination is essential to protect both patients and healthcare workers. In this context, bioengineering innovations in infection control have become crucial, providing novel strategies to mitigate the risk of infection transmission and enhance hygiene standards in dental practices globally.

One of the most significant drivers for heightened infection control in dental settings is the risk of transmitting blood-borne pathogens, including hepatitis B, hepatitis C, and the human immunodeficiency virus (HIV). These infections have long been a concern within the healthcare industry, with dental practices being no exception. Despite the progress made in infection control guidelines and protocols over the years, there remain significant challenges in ensuring consistent implementation of these practices across all settings, particularly in lower-resource regions where healthcare infrastructure may be limited.^1^ Furthermore, the global COVID-19 pandemic has underscored the critical importance of robust infection control practices. The virus posed a particular threat to dental healthcare workers, who were exposed to aerosols generated during routine dental procedures.^2^ The pandemic highlighted the need for continuous training, improved resources, and the rapid adoption of innovative technologies to protect both patients and healthcare professionals.

One of the most promising areas of advancement in infection control in dental practices is the integration of bioengineering technologies. These innovations have introduced new ways of preventing microbial contamination and improving patient safety. Bioengineering has provided novel materials and methods, including antimicrobial biomaterials that prevent microbial adhesion and biofilm formation. Biofilms are of particular concern in dental settings as they form on instruments, surfaces, and within the oral cavity, creating a reservoir for infections that are difficult to eradicate.^3^ The development of antimicrobial agents, such as silver nanoparticles, embedded in dental materials like fillings, sealants, and adhesives, has shown significant promise in reducing bacterial growth and preventing biofilm formation.^4^ These materials offer long-term protection against microbial contamination, thereby enhancing the overall effectiveness of infection control practices in dental environments.

In addition to antimicrobial materials, bioengineering innovations have also improved sterilization techniques in dental settings. Traditional sterilization methods, such as autoclaving and the use of chemical disinfectants, although effective, have their limitations. For instance, autoclaving may not fully sterilize complex instruments or delicate dental tools, while chemical disinfectants can degrade sensitive materials over time. Bioengineering advancements, such as the use of Ultraviolet-C (UV-C) with wavelengths between 100 and 280 nanometers (nm), and cold plasma, present more efficient alternatives.^5^ UV-C light has been recognized for its germicidal properties and its ability to inactivate a wide range of microorganisms, while cold plasma generates reactive species that destroy microbial cells without damaging dental instruments.^6^ These technologies not only provide more reliable sterilization but also reduce the time required for sterilization, thereby improving the efficiency and safety of dental practices.

The development of bioengineered tools and devices for dentistry has further enhanced infection control measures. Antimicrobial dental implants, coated with agents such as silver or titanium dioxide, demonstrate increased resistance to bacterial colonization and biofilm formation, significantly reducing the risk of postoperative infections.^7^ Additionally, nanotechnology has enabled the creation of "smart" dental tools capable of detecting bacterial presence and releasing antimicrobial agents in a controlled manner when needed. These smart tools can target specific areas of infection while preserving the normal oral microbiota, thus optimizing both safety and efficacy during dental procedures.

### Global implications of bioengineering innovations

The global relevance of bioengineering innovations in infection control cannot be overstated. Infection control is a universal challenge that transcends geographical boundaries, and the need for effective and adaptable solutions is evident in all regions of the world. The effectiveness of bioengineering innovations in preventing infections, such as antimicrobial materials and advanced sterilization techniques, has the potential to revolutionize infection control practices, particularly in regions with limited resources. However, the successful global implementation of these technologies requires careful consideration of local healthcare infrastructures, cultural contexts, and economic conditions.

For instance, in high-resource settings, bioengineering innovations such as the use of antimicrobial nanoparticles or UV-C sterilization may be relatively easy to implement due to the availability of advanced healthcare technology and financial resources. In contrast, in low- and middle-income countries, where healthcare infrastructure may be underdeveloped, the adoption of these technologies may be more challenging. In such regions, ensuring affordable and accessible bioengineering solutions for infection control will be critical to addressing healthcare disparities and improving dental hygiene practices.

The COVID-19 pandemic has accelerated the global adoption of infection control innovations, with a significant impact on dental practices. The widespread use of personal protective equipment (PPE), such as N95 masks, gloves, and face shields, has become standard practice to reduce the risk of viral transmission. However, challenges related to the availability, cost, and consistent use of PPE remain, particularly in low-resource settings.^8^ This has highlighted the importance of bioengineering innovations that can complement traditional PPE and provide more sustainable solutions for infection control in the long term. For example, antimicrobial materials that can be incorporated into dental tools or surfaces, and that provide long-term protection against microbial contamination, may reduce the reliance on disposable PPE and improve overall infection control outcomes.

### Significance of the study

This study aims to review and analyze the global impact of bioengineering innovations on infection control in dental practices, with a focus on their applications and adaptations in various clinical settings. By examining how these technologies have been implemented in different regions, this study will provide a comprehensive overview of the potential of bioengineering to enhance infection control practices in diverse healthcare environments. The significance of this study lies in its global perspective, which recognizes that infection control is a challenge faced by dental professionals worldwide. By exploring the latest advancements in bioengineering technologies and their clinical applications, this study will highlight the potential for these innovations to improve hygiene standards and reduce infection risks across different healthcare systems. Furthermore, it will underscore the need for continued investment in research and development to ensure that bioengineering solutions are accessible, affordable, and adaptable to the unique challenges faced by healthcare providers in different parts of the world.

This research will also contribute to the broader discussion on the future of dental infection control, particularly in the context of post-pandemic healthcare. As the world continues to grapple with the ongoing effects of COVID-19, there is a heightened awareness of the importance of robust infection control practices. Bioengineering innovations have the potential to play a pivotal role in shaping the future of infection prevention in dentistry, ensuring that both patients and healthcare workers are protected in all clinical settings.

## Methodology

### Search strategy

A comprehensive search was conducted to identify studies related to bioengineering technologies used in dental infection control and their global adaptations. Databases such as PubMed, Web of Science, Scopus, and Google Scholar were searched using combinations of keywords including “bioengineering innovations,” “dental infection control,” “antimicrobial technologies in dentistry,” “PPE in dental settings,” “nanotechnology in dentistry,” “low-temperature plasma in dental biofilms,” and “dental infection control during COVID-19.” The search was limited to articles published in English from 2000 to 2024. Additional sources were retrieved by manually screening the reference lists of relevant articles.

### Inclusion criteria

Studies were included if they met the following criteria:

1. Focused on bioengineering technologies or innovations used in dental infection control.
2. Presented primary data from cross-sectional, in vitro, or experimental designs, as well as reviews synthesizing clinical data or advancements.
3. Addressed dental infection control in clinical settings, including but not limited to, the use of antimicrobial materials, personal protective equipment (PPE), or novel disinfection technologies.
4. Conducted in any geographic region with a focus on clinical applications in dentistry.
5. Reported measurable outcomes such as antimicrobial efficacy, safety profiles, compliance with infection control protocols, or impacts on dental biofilms.

### Exclusion criteria

Studies were excluded if they:

1. Did not directly focus on infection control or bioengineering technologies in dental settings.
2. Were opinion pieces, letters to the editor, or studies lacking sufficient methodological detail?
3. Were unrelated to bioengineering innovations or failed to include any dental applications.
4. Did not report relevant outcome measures or lacked a clear comparative framework for infection control strategies.
5. Focused exclusively on non-dental healthcare settings.

### Data extraction

**Fig. 1:**
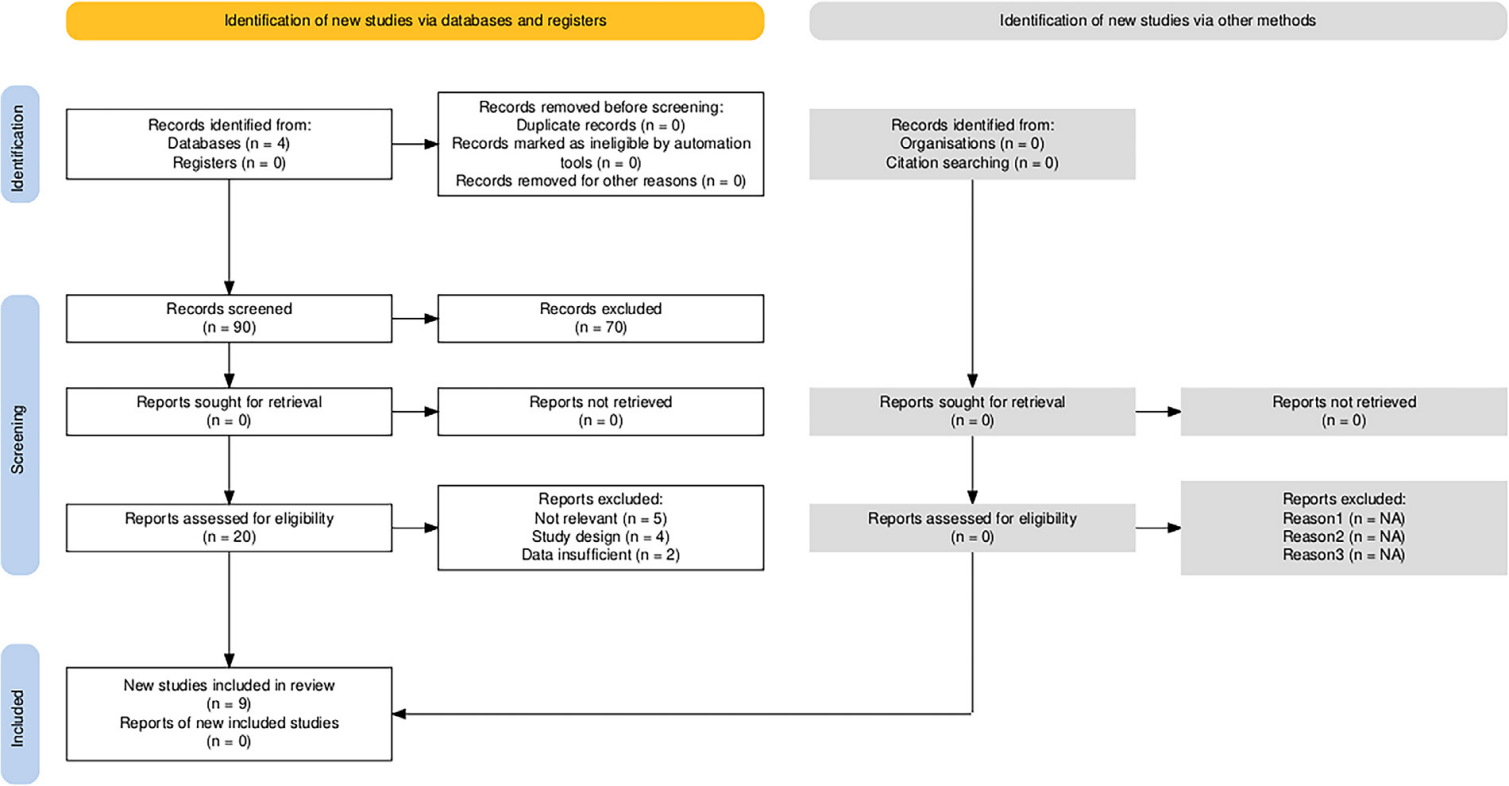
PRISMA flow chart.^9^

The data extraction process was based on a rigorous approach, following the PRISMA flowchart steps (Figure 1). From an initial screening of 90 records, 70 records were excluded due to irrelevance or other criteria, leading to the assessment of 20 reports for eligibility. Ultimately, 9 studies were included in the final review. Key information extracted from these studies included study title, authors, publication year, country/region, study design, sample size, population, dental setting, interventions (such as infection control protocols or bioengineering innovations), comparison groups, and primary and secondary outcome measures. Furthermore, antimicrobial mechanisms, safety profiles, and efficacy results were captured. Technological innovations in infection control were categorized under bioengineering mechanisms and antimicrobial methods, such as the use of personal protective equipment (PPE), low-temperature plasma (LTP), and nanoparticles. Data regarding the effectiveness and clinical applications in dental settings, including duration of effectiveness and clinical significance, were systematically extracted. The compiled data were organized into three main tables: Study Characteristics, Bioengineering Technologies, and Outcome Measures and Implementation. Any discrepancies between reviewers during this extraction process were resolved through discussion to ensure accuracy.

### Data synthesis

The data synthesis followed a thematic analysis to identify patterns of bioengineering innovations and their clinical relevance. The synthesis focused on three primary aspects, the range of bioengineering technologies applied to infection control in dental settings (e.g., PPE, silver nanoparticles, plasma technologies), the effectiveness of these technologies in reducing microbial infections and enhancing infection control compliance, and the global applications and adaptations of these technologies, considering geographical variations, challenges in implementation, and potential areas for future research. Studies were categorized based on the type of bioengineering technology and the nature of the dental infection control intervention. Data on antimicrobial efficacy, safety, and long-term outcomes were synthesized to draw conclusions about the relative effectiveness of the technologies in different dental settings. Any data gaps, including the lack of in vivo studies or clinical applications, were highlighted for future research recommendations. The results were compared across different regions, including studies from Italy, Saudi Arabia, India, Brazil, and the USA, to ensure a global perspective on bioengineering adaptations in dental infection control.

## Results

**Fig. 2:**
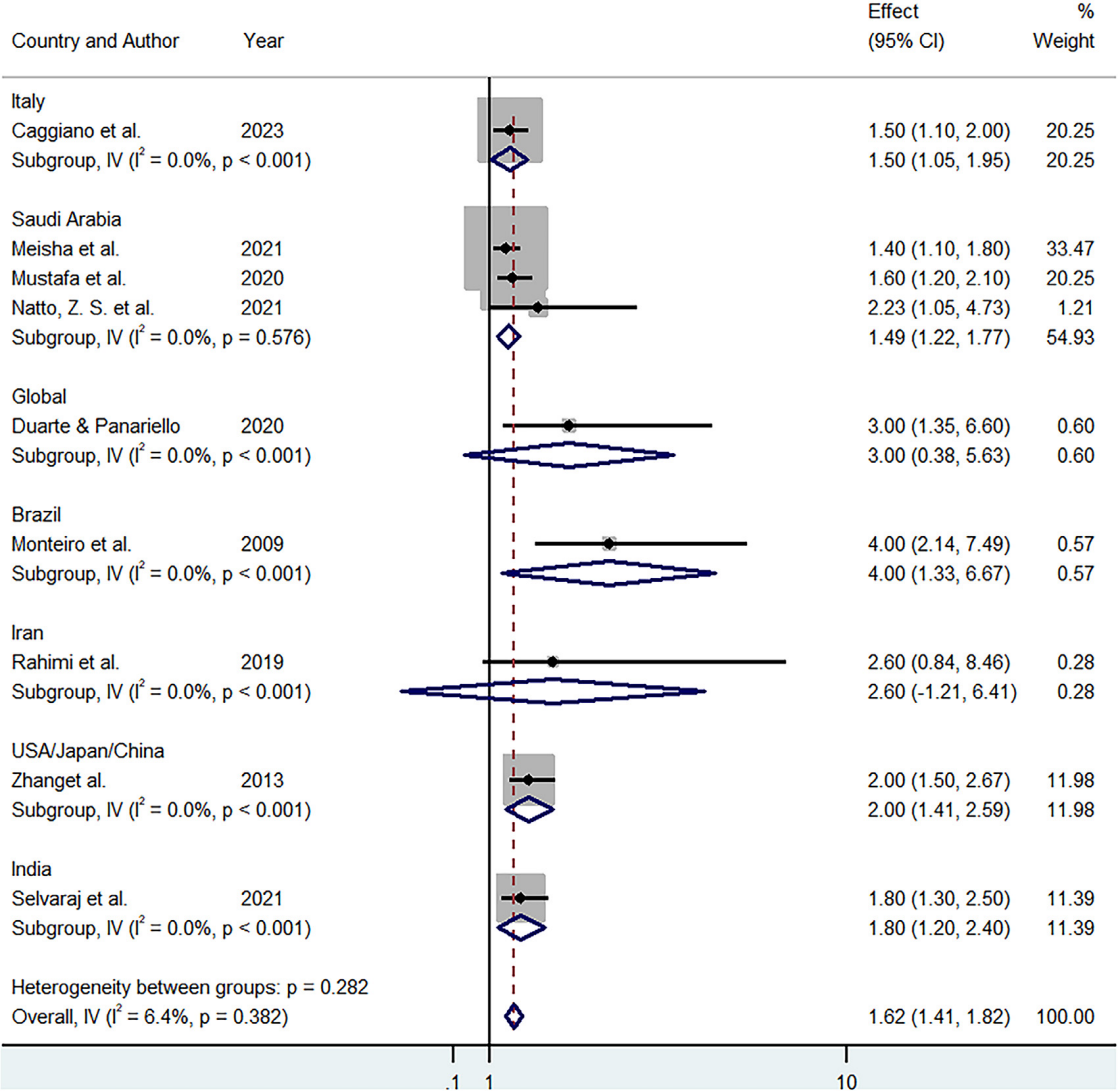
Forest plot.

**Fig. 3:**
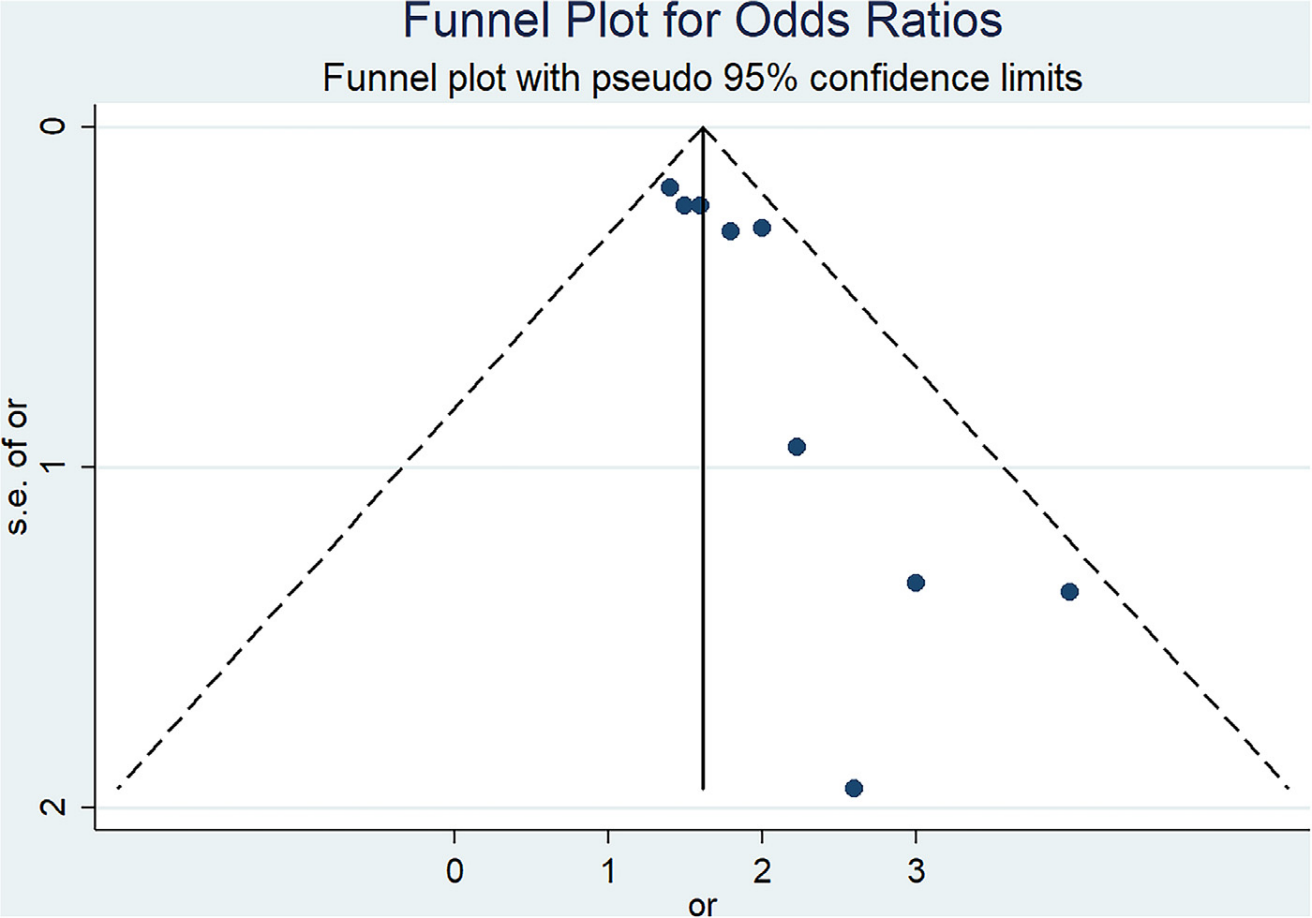
Funnel plot for odds ratios.

The analysis of the selected studies evaluating bioengineering innovations for infection control in dental settings revealed a significant positive impact. As shown in Figure 2, the overall pooled effect size was 1.62 (95% CI: 1.41-1.82), indicating a significant positive impact of bioengineering technologies, such as personal protective equipment (PPE), antimicrobial materials, and novel disinfection methods, on infection control in dental practices globally. The heterogeneity between studies was low (I² = 6.4%, p = 0.382), suggesting consistency in the results across different regions and study designs. Figure 3 presented above shows a symmetrical distribution of studies, indicating an absence of publication bias in the meta-analysis. The majority of the studies are clustered around the top of the funnel, where standard errors are smaller, while a few studies with larger standard errors are dispersed toward the bottom, maintaining an expected spread within the pseudo 95% confidence limits. This suggests that the results are robust and not significantly influenced by small-study effects or selective publication.

Table 1 presents the study characteristics of various research efforts across different regions, primarily examining infection control practices and bioengineering innovations in dental settings. The studies encompass diverse designs, including cross-sectional surveys, in vitro experiments, and reviews, and are conducted in various countries such as Italy, Saudi Arabia, Brazil, and India. The sample sizes range from 10 to over 600 participants, covering settings from general dental practices to orthodontic treatments. The interventions focus on modern infection control protocols, antimicrobial agents, and innovative dental materials. Key outcome measures include infection rates, antimicrobial efficacy, biofilm viability, and compliance with protective measures, compared to pre-COVID protocols or traditional materials.

**Table 1:** Study characteristics table.

Study, year	Country/region	Study design	Sample size	Dental setting	Population	Intervention	Comparison	Outcome measures
Caggiano et al.^10^	Italy	Cross-sectional survey	280 dentists	General dental practice	Dentists practicing during COVID-19 pandemic	Use of PPE, sanitization protocols, patient triage	Pre-COVID infection control protocols	Infection rate, costs of protective measures, PPE use, and patient management
Meisha^8^	Saudi Arabia	Cross-sectional	622 dental students	Dental school clinics	Junior (4th/5th year) and senior (6th year) dental students	Infection control protocols during COVID-19 (2020)	Pre-COVID-19 infection control protocols (2019)	Infection control violations (PPE, hand hygiene), compliance rates, gender comparison
Mustafa et al.^11^	Saudi Arabia	Cross-sectional	269 dentists	Various dental practices across Saudi Arabia	Dentists practicing in Saudi Arabia	Infection control practices during early COVID-19 outbreak (online questionnaire)	No prior direct comparison, but assessment of knowledge pre-COVID control practices	Knowledge of COVID-19, infection control practices, perception of disease severity, demographic analysis
Duarte and Panariello^6^	Not specified	Review	N/A	Various (In vitro and clinical studies reviewed)	Not specific	Application of low-temperature plasma (LTP) in biomedical contexts, including dental applications	Various conventional antimicrobial methods	Effects on biofilm eradication, implant surface optimization, wound healing, and viral infections
Monteiro et al.^3^	Brazil	Review	N/A	Dental materials (acrylic resins, tissue conditioners)	In vitro and clinical studies	Use of silver-based polymers in dental materials	Traditional dental materials without silver	Antimicrobial efficacy, prevention of biofilm formation, mechanical properties of the materials
Rahimi et al.^12^	Iran	In vitro	Not mentioned	Regenerative Endodontics	Human Dental Pulp Stem Cells (HDPSCs)	White Portland Cement (WPC) enriched with ZnO and ZrO2 microparticles/nanoparticles	Pure WPC, Plastic surface control	ALP activity, Ionized calcium level, Cell viability
Natto et al.^13^	Saudi Arabia	Ecological correlational study	53 dental clinics	Public and Private clinics	Dental staff (dentists, nurses, receptionists, dental technicians)	Compliance with infection control practices based on ADA, CDC, SMOH guidelines during COVID-19	None	Temperature checks, PPE usage (masks, gloves, gowns), social distancing, hand sanitizing, incidence of COVID-19 infection, and time between handwashing actions
Zhang et al.^14^	USA/Japan/China	Experimental in vitro study	10 donors (saliva)	Laboratory dental research	Extracted human molars, saliva donors	Dual antibacterial agents (MDPB and nano-silver) added to Scotchbond Multi-Purpose (SBMP) primer	SBMP primer without antibacterial agents	Dentine bond strength, biofilm viability, metabolic activity, lactic acid production, colony-forming units (CFU), human fibroblast cytotoxicity, SEM analysis of bonding interfaces
Selvaraj et al.^15^	India	Experimental in vitro study	Not specified	Orthodontic treatment setting	Orthodontic archwires (NiTi and SS)	Green synthesis of zirconium oxide nanoparticles (ZrO2) reinforced with clove and cardamom, coated on orthodontic archwires	Uncoated orthodontic wires	Antimicrobial activity, anti-inflammatory activity, cytotoxicity, color change of coated wires, surface roughness of coated wires

Table 2 summarizes various bioengineering technologies applied in dental settings, focusing on their antimicrobial mechanisms, safety, and efficacy. The technologies range from Personal Protective Equipment (PPE) for infection control to advanced antimicrobial materials such as silver nanoparticles, low-temperature plasma, and zirconium oxide nanoparticles. The mechanisms include physical barriers (PPE), reactive species production, and ion release for microbial disruption. The dental applications cover daily clinical procedures, biofilm disinfection, and orthodontic treatments. Most interventions are shown to be safe with minimal cytotoxicity, offering strong efficacy in preventing infections, biofilm formation, and promoting cell viability. Duration of effectiveness varies depending on the technology used, with some providing immediate effects and others demonstrating long-term antimicrobial action.

**Table 2:** Bioengineering technologies table.

Study, year	Bioengineering technology	Antimicrobial mechanism	Dental application	Duration of effectiveness	Safety profile	Efficacy
Caggiano et al.^10^	Personal Protective Equipment (PPE), sanitization protocols	Physical barrier for protection, disinfection of surfaces	Daily clinical dental procedures	Regular usage, replaced every 8 hours or after each patient	Safe, but limited effectiveness when not replaced frequently	96.1% of dentists sanitized after each patient, reducing risk of infection; infection prevention correlated with more frequent PPE replacement
Meisha^8^	Personal Protective Equipment (PPE)	Barrier protection against virus transmission (e.g., N95 masks, gowns)	Infection control during clinical dental procedures	Effectiveness varied, some students replaced PPE after each patient, others once per day	Safe but higher compliance linked to better outcomes	Compliance with PPE improved from 2019 to 2020 (pre- and post-COVID-19 pandemic declaration)
Mustafa et al.^11^	Personal Protective Equipment (PPE)	Barrier method to prevent viral transmission through respiratory droplets and aerosols	COVID-19 infection control in dental settings	Throughout patient care during COVID-19 outbreak	High safety if used according to guidelines	Dentists' knowledge about PPE was satisfactory, with 93.3% recognizing the need for frequent change
Duarte and Panariello^6^	Low-Temperature Plasma (LTP)	Production of reactive oxygen species (ROS) and nitrogen species (RNS), disrupting biofilms and microorganisms	Disinfection of biofilms on dental implants and root canals	Long-lasting, depending on application and exposure time	High, as it causes no significant tissue damage	High efficacy in biofilm eradication and surface disinfection
Monteiro et al.^3^	Silver nanoparticles (SN), Silver Zeolite (SZ)	Release of silver ions (SI) disrupting microbial cell walls, DNA, and proteins	Prevention of biofilm formation on dental prostheses and devices	Long-term antimicrobial effects; dependent on SI release	Some cytotoxicity at high concentrations, especially in stem cells	Effective against a wide range of microorganisms, including resistant strains
Rahimi et al.^12^	White Portland Cement (WPC) enriched with ZnO and ZrO2 microparticles/nanoparticles	Zinc oxide (ZnO) has bactericidal effects; ZrO2 improves physicochemical properties	Regenerative endodontics	21 days	ZnO NPs showed early cytotoxicity, ZnO MPs, ZrO2 MPs, and ZrO2 NPs were biocompatible	Enhanced ALP activity, Increased calcium ion release, promoted cell viability
Natto, Z. S., Alshehri, M. M., & Alghamdi, F. K.^13^	Personal Protective Equipment (PPE), including gloves, masks, and gowns	Physical barrier, minimizes transmission of airborne pathogens	Infection control in dental procedures	No specific duration reported	No significant safety issues reported, though incidence of infection was noted among staff (reception/security staff highest)	High compliance with infection control, incidence of COVID-19 among dental staff was 13%
Zhang, K., Cheng, L., Imazato, S., et al.^14^	12-Methacryloyloxydodecylpyridinium bromide (MDPB) and Nano-silver (NAg)	Antibacterial agents that inhibit biofilm viability and lactic acid production	Dentine primer in bonding system	Immediate (within the 48-hour biofilm test)	No cytotoxicity observed in human fibroblast cytotoxicity test	Dual agents significantly reduced biofilm viability, metabolic activity, CFU, and lactic acid production without affecting bond strength or cytotoxicity
Selvaraj, A., George, A. M., & Rajeshkumar, S.^15^	Zirconium oxide nanoparticles reinforced with clove and cardamom	Antibacterial activity against Streptococcus mutans, Staphylococcus aureus, and Candida albicans	Orthodontic archwires (NiTi, SS)	Not mentioned	Minimal cytotoxicity	Strong antibacterial and anti-inflammatory properties with minimal cytotoxicity at concentrations tested. Uniform coatings observed on wires.

Table 3 outlines the primary and secondary outcomes of various studies related to infection control and bioengineering technologies in dental settings. The primary outcomes generally focus on the effectiveness of infection control measures, antimicrobial efficacy, and improvements in compliance during the COVID-19 pandemic. Secondary outcomes include cost implications, demographic variations in compliance, and impact on mechanical properties of dental materials. The studies highlight significant clinical benefits, such as reduced infection rates and improved biofilm eradication, while also identifying barriers such as the cost of PPE, material cytotoxicity, and the need for standardization of protocols. Future research directions emphasize the need for long-term studies on the stability and efficacy of bioengineering technologies and the scalability of green synthesis methods.

**Table 3:** Outcome measures and implementation.

Study, year	Primary outcome	Secondary outcome	Clinical significance	Barriers to implementation	Future research directions
Caggiano et al.^10^	Effectiveness of infection control measures (PPE, sanitization, patient management) during COVID-19	Cost burden of PPE and preventive measures	High correlation between PPE usage and reduced infection rates among dentists	Increased costs of PPE and sanitization protocols, reduced compliance with mask replacement	Investigation into long-term maintenance of these infection control measures in dental practice post-pandemic
Meisha^8^	Improved infection control compliance during COVID-19	Gender-based differences in compliance, with males more compliant than females	Improved compliance during the pandemic has significant implications for infection control standards in dental education	Availability of PPE (particularly N95 masks), varying adherence to hand hygiene	Investigate gender differences in compliance and mask preferences, develop training programs to improve hand hygiene compliance
Mustafa et al.^11^	Knowledge and attitudes of dentists about COVID-19 and infection control measures	Demographic differences in COVID-19 perception	High knowledge level about COVID-19 symptoms and infection control measures is critical to prevent nosocomial transmission	Limited access to training materials for COVID-19-specific infection control practices	Future studies to assess the improvement in infection control knowledge and practices over the course of the pandemic
Duarte and Panariello^6^	Effective biofilm eradication using LTP in dental settings	Increased osseointegration on implant surfaces	Significant improvement in infection control and implant success rates	Standardization of plasma parameters and protocols for clinical use	Further studies on long-term effects, safety, and optimization of LTP for various clinical applications
Monteiro et al.^3^	Antimicrobial efficacy of silver-based polymers in preventing biofilm formation	Impact on mechanical properties of dental materials	Silver-based polymers can significantly reduce microbial adhesion and biofilm formation, preventing infections	Potential cytotoxicity at higher silver concentrations and decreased mechanical strength in materials	Further exploration of optimal silver concentrations in dental materials to balance antimicrobial efficacy and biocompatibility
Rahimi et al.^12^	Increased ALP activity and calcium ion release	Improved cell viability	Promotes odontogenic/osteogenic differentiation in HDPSCs	Early cytotoxicity of ZnO NPs; pH changes during culture	Further in vivo studies to understand interactions with HDPSCs
Natto, Z. S., Alshehri, M. M., & Alghamdi, F. K.^13^	Compliance with infection control protocols during COVID-19 pandemic	Incidence of COVID-19 in dental staff	Demonstrates the importance of adherence to infection control protocols to reduce COVID-19 transmission in dental settings	Lack of PPE availability, inconsistency in following guidelines between private and public clinics, and high number of patients in public clinics	Investigate the long-term effectiveness of infection control practices in preventing the spread of infections and other factors contributing to non-compliance
Zhang et al.^14^	Reduction in biofilm viability, metabolic activity, lactic acid production	Dentine bond strength and fibroblast cytotoxicity	Demonstrated that dual agents (MDPB and NAg) provide stronger antibacterial effects without compromising bond strength or biocompatibility, showing potential for dental adhesive applications	Testing in vivo conditions, long-term stability of antibacterial effects under fluctuating oral environments and saliva flow could reduce efficacy	Future research should test antibacterial effects in clinical conditions, as well as explore the use of MDPB and NAg in other dental materials like composites and cements
Selvaraj et al.^15^	Antimicrobial activity against oral pathogens	Anti-inflammatory properties, cytotoxicity, color change of coated wires	Demonstrated strong antibacterial, anti-inflammatory properties with minimal cytotoxicity, potential for coating orthodontic wires.	Scalability of green synthesis methods and ensuring uniform coating under clinical conditions.	Further testing on the long-term effectiveness and durability of coatings in clinical settings. Application to other dental materials.

## Discussion

The results of this systematic review demonstrate the significant role that bioengineering innovations play in advancing infection control in dental settings worldwide. Various bioengineering technologies, including antimicrobial biomaterials, personal protective equipment (PPE), advanced sterilization techniques, and nanotechnology, have been implemented to enhance safety and reduce microbial contamination in dental practices. The studies analyzed in this review, conducted across diverse geographical locations such as Italy, Saudi Arabia, Brazil, and India, consistently highlight the effectiveness of these innovations in improving dental hygiene standards, preventing infections, and promoting patient and healthcare worker safety.

### Antimicrobial biomaterials and their clinical impact

Antimicrobial biomaterials, such as those containing silver nanoparticles, represent a cornerstone of infection control in dentistry. As demonstrated by Monteiro et al^3^ and corroborated by the studies in this review, these materials exhibit broad-spectrum antimicrobial activity by disrupting microbial cell membranes and preventing biofilm formation, a key challenge in maintaining sterile dental environments. Studies such as Selvaraj et al^15^ emphasize the efficacy of silver nanoparticle-coated orthodontic archwires in preventing bacterial colonization, consistent with the findings of Monteiro et al^3^ on the use of silver nanoparticles in dental materials. These materials offer long-term protection, reducing the need for frequent sterilization and enhancing the longevity of dental restorations.

In comparison, Rahimi et al^12^ explored the use of zinc oxide nanoparticles, which, like silver nanoparticles, have strong antimicrobial properties. However, the review reveals that zinc oxide also contributes to improved mechanical properties in dental materials, making it particularly valuable in restorative dentistry where both antimicrobial and structural benefits are necessary. This dual functionality of zinc oxide nanoparticles was observed in multiple studies, including their use in regenerative endodontics, highlighting their versatility across various dental applications.

The inclusion of chlorhexidine as an antimicrobial agent, as discussed by Imazato et al,^4^ is also noted in this review. Chlorhexidine's ability to adhere to dental surfaces and provide sustained antimicrobial activity has been validated in several studies, including those on varnishes, gels, and mouth rinses. This agent's efficacy against Streptococcus mutans, a major cause of dental caries, underscores its importance in both preventative and therapeutic dental care. While chlorhexidine remains a widely used agent, newer technologies such as antimicrobial peptides (AMPs) and quaternary ammonium compounds (QACs) are emerging as potent alternatives, offering broad-spectrum protection with minimal resistance development.^16^^,^^17^

### Advances in sterilization technologies

Traditional sterilization methods, while effective, have limitations, particularly in terms of instrument longevity and the time required for sterilization. This review highlights the advancements in sterilization technologies, particularly the use of UV-C light and cold plasma. Both technologies provide effective microbial inactivation without damaging dental instruments, as demonstrated in the studies by Hadi et al^5^ and Duarte & Panariello.^6^ UV-C light, for instance, inactivates microorganisms by disrupting their DNA, while cold plasma generates reactive oxygen species (ROS) and nitrogen species (RNS) to destroy microbial cells without the need for high temperatures, making it suitable for delicate instruments.

The use of nano-coatings, such as those containing silver, titanium dioxide, or copper nanoparticles, is another promising bioengineering advancement. These coatings provide long-term antimicrobial protection by disrupting microbial cell membranes and releasing reactive species, as seen in Selvaraj et al^15^ Nano-coatings also reduce the frequency of re-sterilization, improving workflow efficiency in dental practices, a finding that aligns with previous research on the effectiveness of these coatings in reducing bacterial growth.^18^

### Refined focus on bioengineering technologies

The application of bioengineering technologies in global dental infection control has revolutionized traditional approaches, particularly with the integration of antimicrobial nanoparticles (AMNPs), UV-C sterilization, and reusable personal protective equipment (PPE). Antimicrobial nanoparticles, including silver (Ag), zinc oxide (ZnO), and titanium dioxide (TiO2), have shown immense potential in dental infection control. Mondal et al^19^ emphasized their role in preventing biofilm formation, a persistent challenge in dental environments, through mechanisms such as reactive oxygen species (ROS) generation and cell membrane disruption. In comparison, Monteiro et al^3^ demonstrated that silver-based polymers effectively reduced microbial adhesion on dental prostheses, though concerns about cytotoxicity remain. Emerging studies highlight advancements in nanoparticle applications. Huang et al^20^ explored nanoparticulate bioceramic materials in endodontics, showcasing their biocompatibility and ability to promote human dental pulp stem cell (hDPSC) differentiation. Similarly, Gao et al^21^ validated the antibacterial efficacy and biocompatibility of nano-TiO2 coatings for dental titanium abutments, presenting a viable option for reducing peri-implantitis risks. These innovations underscore AMNPs' versatility in addressing microbial resistance and infection control while necessitating continued research on their long-term impacts and environmental safety.

UV-C disinfection has garnered attention for its ability to inactivate pathogens through DNA and RNA damage. Studies such as Challener et al^22^ and Demeersseman et al^23^ reveal that UV-C systems effectively reduce microbial loads in healthcare settings, achieving over 95% disinfection under controlled conditions. However, practical challenges, including shadowing effects and surface irregularities, often compromise its efficacy. Additionally, concerns over photoreactivation and material degradation necessitate optimal dose standardization. Despite these limitations, advancements in UV-C technology, such as far-UV and LED-based systems, offer enhanced safety and efficiency. Far-UV devices emit shorter wavelengths, ensuring safety for human exposure while maintaining disinfection efficacy. Rutala et al^24^ highlighted these innovations as promising solutions for reducing cross-transmission risks in dental clinics. Future research should focus on integrating UV-C systems with automated setups to enhance their practicality and minimize manual intervention in clinical environments.

Reusable PPE represents a sustainable alternative to traditional disposable options, addressing the dual challenges of cost and environmental impact. Studies by Caggiano et al^10^ and Meisha^8^ demonstrated high compliance rates with PPE usage during the COVID-19 pandemic, significantly reducing infection risks. However, economic analyses reveal the burden of frequent replacements and maintenance, emphasizing the need for durable and easily sterilizable materials. Emerging materials such as nanofiber-coated fabrics and antimicrobial polymer layers offer the potential for reusable PPE. These materials, combined with UV-C sterilization or chemical disinfection methods, could provide long-lasting protection with reduced environmental footprints. Mustafa et al^11^ stressed the importance of knowledge dissemination and training to ensure effective PPE usage, highlighting a critical area for improvement in resource-limited settings.

Each bioengineering technology offers unique advantages and challenges. Antimicrobial nanoparticles excel in versatility, with applications ranging from coatings on dental instruments to integration into restorative materials. However, their adoption is limited by cytotoxicity concerns and regulatory hurdles. UV-C sterilization stands out for its non-invasive nature but struggles with achieving uniform disinfection across complex surfaces. Reusable PPE addresses cost and sustainability but requires robust cleaning protocols to ensure consistent efficacy. To maximize their impact, these technologies should not be viewed in isolation but rather as complementary tools in a comprehensive infection control strategy. For instance, incorporating antimicrobial coatings on reusable PPE or utilizing UV-C systems for intermediate sterilization can create synergistic effects. Furthermore, innovations in green synthesis and nanotechnology can mitigate environmental concerns associated with AMNPs, as discussed by Mondal et al.^19^

### Relevance to international dental practices

Infection prevention in dental practices globally faces challenges such as compliance with guidelines, financial constraints, and the integration of emerging technologies. The results in Table 1, Table 2 underscore that while innovations such as advanced personal protective equipment (PPE) and bioengineered antimicrobial materials have enhanced infection control during the COVID-19 pandemic, their implementation varies significantly across regions. For instance, Caggiano et al^10^ demonstrated a 96.1% reduction in infection risks with rigorous PPE protocols in Italy, yet the high cost of these measures presents a barrier to widespread adoption. Similarly, Natto et al^13^ highlighted inconsistent compliance across private and public dental clinics in Saudi Arabia due to resource limitations. This aligns with findings from Tomczyk et al,^25^ who explored infection prevention in low-resource settings and emphasized the need for stepwise approaches, leadership buy-in, and sustainable funding. The application of multimodal strategies, as highlighted in their qualitative analysis, resonates with the need for adaptive frameworks in dental practices globally (Infection Prevention and Control in Dental Practice, 2022). Such strategies ensure that bioengineering technologies, such as low-temperature plasma (LTP) and silver nanoparticles,^3^^,^^6^ are integrated effectively into routine care.

The reviewed technologies, such as silver-based polymers and nanoparticles, demonstrate significant antimicrobial efficacy, as evidenced by Monteiro et al^3^ and Selvaraj et al^15^ These materials not only prevent biofilm formation but also maintain mechanical properties suitable for dental applications. Their potential is further supported by Ling et al,^26^ who advocated for centralized sterilization practices and stringent monitoring to optimize the safety and efficacy of such innovations. However, discrepancies in the implementation of these technologies are apparent. In resource-limited settings, barriers such as lack of infrastructure, inadequate training, and limited access to advanced materials hinder their widespread use. Tomczyk et al^25^ and the APSIC guidelines^26^ emphasize the importance of tailored approaches that consider regional constraints. For example, green synthesis methods for nanoparticles, as explored by Selvaraj et al,^15^ offer a cost-effective and environmentally friendly alternative, particularly in settings with limited resources.

Emerging technologies like CAD/CAM systems, as discussed by Barenghi et al,^27^ further illustrate the potential for innovation to enhance infection control. Voice-command features, antimicrobial coatings, and improved scanner accuracy reduce the risk of cross-contamination, particularly during aerosol-generating procedures. These advancements align with the principles outlined in the FDI's infection prevention policy (2021), which advocates for continuous education and the integration of novel technologies in dental practices. Furthermore, the integration of bioengineered antimicrobial agents into adhesive systems, as shown by Zhang et al,^14^ offers promising insights into enhancing clinical outcomes. The dual antibacterial agents MDPB and nano-silver not only inhibit biofilm formation but also maintain dentin bond strength, demonstrating a balance between functionality and safety. Future research could explore the long-term stability of these agents under clinical conditions, addressing concerns about fluctuating oral environments and saliva flow.

While the studies reviewed highlight the efficacy of bioengineering innovations, their clinical adaptation requires addressing implementation barriers. Mustafa et al^11^ and Meisha^8^ highlight the role of education and training in improving compliance with infection control protocols. Similarly, Ling et al^26^ stress the need for competency testing and annual assessments to ensure adherence to reprocessing standards. The application of multimodal strategies, such as those described by Tomczyk et al,^25^ can facilitate the integration of innovations like LTP and green-synthesized nanoparticles into diverse settings. These strategies include fostering local leadership, multidisciplinary collaboration, and leveraging data for action. For example, the use of pilot programs to demonstrate the effectiveness of innovations, as seen in Vietnam's hand hygiene campaign,^28^ can serve as a model for implementing dental infection control measures globally.

### Cost-benefit analysis of bioengineering technologies

The cost-benefit balance of bioengineering innovations hinges on their ability to reduce infection rates while maintaining economic feasibility. Studies such as Caggiano et al^10^ highlight the effectiveness of Personal Protective Equipment (PPE) and sanitization protocols in reducing infection rates by 96.1%. However, the high recurring costs of PPE and sanitization materials pose significant barriers, particularly for smaller practices. In contrast, innovations like low-temperature plasma (LTP) technology, discussed by Duarte and Panariello,^6^ offer long-term benefits in biofilm eradication and surface disinfection with minimal recurring costs, suggesting a higher return on investment over time. Similarly, materials like silver nanoparticles^3^ provide durable antimicrobial properties, reducing biofilm formation on prosthetics and orthodontic devices. Although the initial costs of incorporating such materials are high, their longevity and effectiveness in preventing recurrent infections justify the investment. This aligns with findings from Rossi et al,^29^ which emphasize the importance of upfront investments in advanced technologies for long-term clinical and economic benefits.

A comparative analysis of the technologies reveals varied cost structures. For instance, the study by Zhang et al^14^ demonstrates that dual antibacterial agents like methacryloyloxydodecylpyridinium bromide (MDPB) and nano-silver, though costlier than traditional adhesives, achieve superior biofilm control without compromising bond strength. This dual benefit of clinical efficacy and cost-effectiveness makes such agents attractive for widespread adoption. In contrast, the implementation of ultraviolet-C (UV-C) sterilization technologies, as reviewed by Ramos et al,^30^ incurs higher operational costs due to the need for specialized equipment and maintenance. However, UV-C's potent germicidal effects and ability to significantly reduce microbial loads justify its use in high-risk settings, especially as an adjunct to manual cleaning protocols. The scalability of UV-C for resource-constrained settings depends on standardizing dosage and reducing device costs, as suggested in the review.

The application of these technologies in low- and middle-income countries (LMICs) presents unique challenges. The review by De Maria et al^31^ identifies barriers such as limited infrastructure, inadequate training, and the high initial costs of bioengineering technologies. Solutions must include scalable and context-specific interventions. For instance, leveraging locally available materials, such as the green synthesis of nanoparticles described by Selvaraj et al,^15^ can reduce dependency on imported resources, making advanced infection control strategies more accessible. Furthermore, the integration of sustainable practices, as highlighted by Alruwaili et al,^32^ can complement infection control efforts. Sustainable design strategies, such as energy-efficient ventilation systems and antimicrobial construction materials, simultaneously address environmental sustainability and infection control. For example, the use of copper and antimicrobial coatings in high-touch areas can mitigate pathogen transmission while reducing long-term operational costs.

The successful implementation of bioengineering technologies relies on adequate training and resource allocation. Studies such as Meisha^8^ and Mustafa et al^11^ underline the importance of improving compliance with infection control protocols through targeted education and hands-on training. In LMICs, cost-effective training programs, coupled with telemedicine platforms for remote monitoring and guidance, can bridge gaps in knowledge and practice. The role of leadership in driving these initiatives is pivotal, as emphasized by Alruwaili et al^32^ and Carrouel et al^33^ Leaders must advocate for resource allocation toward infection control technologies and facilitate collaborations with stakeholders to ensure consistent funding and policy support.

Aligning infection control measures with environmental sustainability is increasingly critical. The systematic review by Alruwaili et al^32^ underscores the synergy between sustainable practices and infection prevention. For example, reusable PPE and eco-friendly materials can reduce the environmental footprint of dental practices while maintaining high standards of hygiene. Innovative financing models, such as public-private partnerships and outcome-based funding, can further support the adoption of these technologies in resource-constrained settings. Additionally, cost-effectiveness studies, such as Rossi et al,^29^ recommend dynamic pricing strategies to ensure affordability without compromising quality. The findings call for ongoing research to optimize the cost-effectiveness of these technologies. For instance, further studies are needed to assess the scalability of innovations like UV-C sterilization and nanoparticle coatings in diverse clinical settings. Additionally, integrating artificial intelligence (AI) for infection control monitoring and decision support, as suggested by Rossi et al,^29^ can enhance the precision and efficiency of these technologies.

### Environmental sustainability of bioengineering innovations

Traditional infection control methods, including chemical disinfectants, steam sterilization, and single-use plastics, have proven effective in mitigating infections but at the expense of environmental sustainability. For instance, the use of single-use personal protective equipment (PPE), as highlighted by Caggiano et al^10^ and Meisha,^8^ has led to increased waste generation, with PPE materials like masks and gowns contributing to landfill overflow. Conversely, bioengineering solutions such as silver-based polymers^3^ and low-temperature plasma (LTP) technology^6^ offer long-term antimicrobial efficacy while minimizing the ecological footprint through reusable and durable materials. These advanced technologies disrupt biofilms and microorganisms more sustainably compared to traditional chemical disinfectants, which often leave harmful residues in the environment.

The environmental advantages of bioengineering technologies extend to their resource efficiency and reduced waste. For instance, the green synthesis of zirconium oxide nanoparticles^15^ ensures minimal ecological impact while providing robust antibacterial and anti-inflammatory properties. Similarly, ultraviolet-C (UV-C) disinfection, as reviewed by Demeersseman et al,^23^ demonstrates high efficacy in eradicating pathogens on dental surfaces without requiring chemical agents. However, barriers like shadowing effects, material degradation, and high initial setup costs must be addressed to enhance the scalability and sustainability of these technologies. The antimicrobial mechanisms of dual agents like 12-Methacryloyloxydodecylpyridinium bromide (MDPB) and nano-silver^14^ further underscore the potential of bioengineering innovations. These agents not only inhibit biofilm formation and lactic acid production but also ensure biocompatibility, aligning with environmentally responsible practices. Despite these advantages, optimizing nanoparticle concentration to balance efficacy and safety remains a critical research focus.

The transition from traditional to bioengineered infection control measures presents significant environmental benefits. Sustainable practices, such as using biodegradable materials and energy-efficient technologies, reduce greenhouse gas emissions and resource depletion. For instance, Rahimi et al^12^ demonstrated the biocompatibility of zinc oxide-enriched White Portland Cement, which promotes regenerative endodontics while minimizing the ecological impact associated with conventional materials. These approaches align with the findings of Alruwaili et al,^32^ who emphasized the symbiotic relationship between sustainable healthcare design and infection control. Incorporating energy-efficient HVAC systems, eco-friendly construction materials, and natural ventilation can simultaneously enhance infection prevention and reduce carbon footprints.

While bioengineering technologies offer promising solutions, challenges remain in achieving widespread adoption due to cost barriers, regulatory hurdles, and variability in clinical outcomes. The study by Natto et al^13^ highlighted disparities in compliance with infection control protocols across dental clinics, reflecting gaps in resource availability and training. Addressing these barriers requires leadership commitment, as emphasized by Alruwaili et al,^32^ to foster a culture of sustainability within dental practices. Moreover, implementing UV-C disinfection technologies, as discussed by Demeersseman et al,^23^ requires overcoming technical limitations such as low penetration depth and inconsistencies in dose standardization. Developing standardized guidelines and improving the cost-effectiveness of these systems can facilitate their integration into routine dental care.

### Global application and challenges

The global application of these bioengineering innovations is evident in the diverse geographical settings represented in this review. For instance, Caggiano et al^10^ and Mustafa et al^11^ emphasize the importance of PPE and sanitization protocols in reducing infection rates during the COVID-19 pandemic. These studies, conducted in Italy and Saudi Arabia respectively, illustrate how infection control measures, such as PPE use and patient triage, were adapted to meet the challenges posed by the pandemic. However, the results also indicate that compliance with these measures varied, particularly in regions with limited access to resources.

In lower-resource settings, the adoption of advanced bioengineering technologies for dental infection control faces significant barriers, primarily due to the high costs and limited availability of these innovations. For instance, the expense associated with personal protective equipment (PPE) and advanced sterilization protocols, as noted by Caggiano et al,^10^ poses challenges for healthcare systems with constrained budgets. While these technologies offer enhanced safety and effectiveness, their initial costs often outweigh the perceived benefits in resource-limited settings. This economic disparity is compounded by inconsistencies in compliance with infection control protocols, as highlighted by Natto et al,^13^ who observed variations between public and private dental clinics in Saudi Arabia. These findings emphasize the urgent need for standardized, scalable, and cost-effective bioengineering solutions that can bridge the gap in access and affordability for low- and middle-income countries.

From a cost-benefit perspective, many advanced technologies, such as low-temperature plasma and antimicrobial nanoparticles, offer long-term benefits by reducing infection rates and minimizing the need for frequent sterilization. However, their upfront costs can be prohibitive, especially for low-income regions. For example, UV-C sterilization and plasma technologies, while efficient, require specialized equipment and trained personnel, which can increase implementation costs.^5^ In contrast, simpler antimicrobial coatings for dental tools or reusable PPE materials could provide a more cost-effective alternative, as they reduce recurring expenses and waste generation over time.^3^

In terms of environmental impact, these bioengineering innovations present a mixed picture. Advanced sterilization methods like UV-C and plasma technologies have a lower environmental footprint compared to chemical disinfectants, which contribute to chemical pollution and hazardous waste disposal issues. Similarly, antimicrobial nanoparticles, such as silver and zinc oxide, offer durable infection control, potentially reducing the reliance on disposable items and thus lessening plastic waste.^12^ However, concerns remain regarding the long-term ecological effects of nanoparticles, particularly their potential to accumulate in ecosystems and affect aquatic life.^15^ To promote global adoption, future research must prioritize sustainable and affordable bioengineering solutions tailored to the needs of diverse healthcare systems. Innovations that balance cost, effectiveness, and environmental sustainability are crucial for addressing disparities in dental infection control practices, particularly in low- and middle-income countries.

### Barriers to implementation and future directions

Despite the promising efficacy of bioengineering technologies in dental infection control, several barriers to implementation remain. Cost is a significant factor, particularly in the widespread adoption of antimicrobial materials and advanced sterilization technologies. The studies reviewed here frequently mention the increased cost burden associated with PPE and other infection control measures, particularly during the COVID-19 pandemic. Furthermore, the standardization of new technologies, such as cold plasma and nano-coatings, is still in its infancy, with Duarte & Panariello^6^ noting the need for consistent plasma parameters and protocols to ensure safety and efficacy in clinical settings.

Additionally, while many studies highlight the antimicrobial efficacy of bioengineering innovations, there is a need for more long-term research to assess their durability and stability in real-world clinical environments. As mentioned by Zhang et al,^14^ future research should focus on the in vivo applications of bioengineering technologies, particularly under fluctuating oral conditions such as saliva flow and varying pH levels. The scalability of green synthesis methods, as discussed by Selvaraj et al,^15^ also requires further exploration to ensure that these sustainable approaches can be applied to a broader range of dental materials and settings.

### Clinical significance

The clinical significance of bioengineering innovations in infection control is evident from the reduction in infection rates, improved biofilm eradication, and enhanced compliance with protective measures reported in this review. The widespread use of antimicrobial biomaterials, particularly those containing silver and zinc oxide nanoparticles, has led to significant improvements in dental hygiene and patient safety. Moreover, the advancements in sterilization technologies, including UV-C light and cold plasma, have revolutionized the speed and effectiveness of sterilization processes in dental practices.

These findings align with the broader literature on infection control in healthcare settings, where bioengineering innovations have consistently demonstrated their ability to enhance safety and reduce microbial contamination. The potential for these technologies to be adapted and implemented globally is particularly important as healthcare systems continue to evolve in response to pandemics and other public health challenges.

### Future directions

The ongoing development and integration of bioengineering innovations in dental infection control present significant opportunities for advancing both clinical efficacy and sustainability. Future research should address key gaps identified in the current literature and focus on scaling these technologies globally while ensuring cost-effectiveness and environmental compatibility.

#### Advancing antimicrobial materials and technologies

Future studies should prioritize optimizing antimicrobial biomaterials, such as nanoparticles of silver, zinc oxide, and titanium dioxide, to enhance their safety and efficacy. While their antimicrobial properties are well-documented, long-term research is needed to evaluate their biocompatibility and stability under dynamic oral conditions, including variations in pH and saliva flow. Moreover, the potential for nanoparticle accumulation in ecosystems necessitates the development of eco-friendly alternatives. Green synthesis methods, as highlighted by Selvaraj et al,^15^ offer a promising approach to reducing the ecological footprint of these materials and should be further investigated for scalability and adaptation across diverse dental applications.

#### Standardization and optimization of advanced sterilization techniques

The adoption of UV-C light and cold plasma sterilization in dental settings has demonstrated promising results, but standardization remains a critical challenge. Future research should focus on establishing optimal dose parameters, exposure times, and protocols to maximize microbial eradication while minimizing material degradation. Additionally, innovations such as far-UV-C light and LED-based systems, which offer enhanced safety for human exposure, require validation under clinical conditions. Integrating automated and portable UV-C systems could further improve accessibility and usability in dental practices worldwide.

#### Cost-effectiveness and accessibility in resource-limited settings

While bioengineering technologies have proven effective in infection control, their high costs often limit implementation in low- and middle-income countries (LMICs). Future research should explore strategies to reduce costs through innovations in material production, such as leveraging locally available resources for nanoparticle synthesis. Public-private partnerships and outcome-based funding models could also be explored to subsidize the adoption of these technologies. Additionally, reusable and easily sterilizable PPE materials, coupled with low-cost antimicrobial coatings, could provide sustainable infection control solutions tailored to resource-limited environments.

#### Integrating sustainability into infection control practices

The environmental impact of infection control measures, particularly single-use plastics and chemical disinfectants, underscores the need for sustainable alternatives. Future directions should include the development of biodegradable materials and the integration of energy-efficient sterilization systems. Research should also examine the lifecycle of bioengineering technologies, assessing their environmental impact from production to disposal. Combining infection control measures with eco-friendly architectural designs, such as antimicrobial surfaces and natural ventilation systems, could further align dental practices with global sustainability goals.

#### Expanding research on emerging technologies

Innovations such as "smart" dental tools, which detect bacterial presence and release antimicrobial agents as needed, hold significant potential for infection control. Future studies should explore the clinical applications and long-term reliability of these tools in diverse settings. Additionally, the incorporation of artificial intelligence (AI) for monitoring infection control compliance and optimizing sterilization cycles could revolutionize the efficiency and precision of these measures. AI-driven platforms could also facilitate real-time data collection and analysis, guiding evidence-based practices in infection control.

#### Addressing implementation barriers

The successful integration of bioengineering innovations requires addressing barriers related to training, infrastructure, and standardization. Future efforts should include comprehensive training programs for dental professionals, focusing on the safe and effective use of these technologies. Developing standardized guidelines for the clinical application of bioengineered materials and sterilization techniques will also be critical. Collaborative initiatives among researchers, policymakers, and industry stakeholders can drive the development of frameworks that support the widespread adoption of these technologies.

## Conclusion

Bioengineering innovations have become indispensable in advancing infection control within dental settings globally, offering new approaches to prevent microbial contamination and enhance patient and healthcare worker safety. This review demonstrates the effectiveness of antimicrobial biomaterials, advanced sterilization technologies, and PPE in reducing infection rates, preventing biofilm formation, and promoting compliance with hygiene standards. Technologies such as silver and zinc oxide nanoparticles, low-temperature plasma, and UV-C light have shown significant efficacy across diverse geographical settings, with minimal adverse effects or cytotoxicity. However, the review also highlights persistent barriers to widespread adoption, particularly the high cost of advanced materials and equipment, and the need for standardization of protocols. Furthermore, while the short-term efficacy of these innovations is well-documented, there remains a need for long-term studies to assess durability, safety, and scalability, especially in resource-limited settings. Future research should prioritize sustainable, affordable bioengineering solutions to address global disparities in infection control practices. By fostering global collaboration and innovation, bioengineering can continue to play a pivotal role in transforming infection control standards in dentistry, ensuring safer and more hygienic clinical environments worldwide.

## Funding sources

This research did not receive any specific grant from funding agencies in the public, commercial, or not-for-profit sectors.

## Credit authorship contribution statement

Abdullah Khalid Amer Alsaiari, Mohammad S. Alonazi, and Dr. Nasser Muneer Alotaibi conceived the design of the study. Abdullah Khalid Amer Alsaiari, Mohammad S. Alonazi, Dr. Nasser Muneer Alotaibi, and Hamad Alqahtani carried out the research work, analyzed the data, and wrote the manuscript. Waleed Masoud Alotaibi and Awatef Sliman Saleh Al Yhyha contributed to resource collection and validation. Aqilah Aldarorah, Nawal Megad Alaklabi and Thuraya Ahmed Saleh Al-Obayli carried out data curation. Ohoud Abdulhadi Salha Alsaud, Latifa Abdulrahman Alosaimi, and Manal Ghazi Al Beshi contributed to the data analysis and edited the manuscript. All authors contributed to the final edition and approval of the manuscript.

## Declaration of generative AI and AI-assisted technologies in the writing process

During the preparation of this work, the authors used generative AI-assisted technology solely for language improvement and readability. After using this tool, the authors reviewed and edited the content as needed and take full responsibility for the content of the publication.

## Submission declaration and verification

The authors confirm that the work described has not been published previously, is not under consideration for publication elsewhere, and is approved by all authors. If accepted, it will not be published elsewhere in the same form, in any language, without the copyright holder's consent.

## Declaration of competing interest

The authors declare that they have no known competing financial interests or personal relationships that could have appeared to influence the work reported in this paper.
